# Unsuccessful Self-Enucleation in a Schizophrenic Patient

**DOI:** 10.1155/2014/237214

**Published:** 2014-09-21

**Authors:** Noam Bar-Yaakov, Romi Noy Achiron, Michael Paul, Asaf Achiron

**Affiliations:** ^1^The Ruth and Bruce Rappaport Faculty of Medicine, Technion Institute of Technology, Haifa, Israel; ^2^Tel Aviv University, Tel Aviv, Israel; ^3^Department of Ophthalmology, The Edith Wolfson Medical Center, Holon, Israel

## Abstract

Self-enucleation is a very unusual form of self-mutilation directly linked to mental illness. In this case we present a 26-year-old schizophrenic patient who attempted to enucleate his eye with a rollerball pen. Antipsychotic therapy and emergency surgery saved the patient eye and emphasize the importance of quick response and good collaboration between psychiatric and ophthalmic teams.

## 1. Introduction

Self-enucleation, or “oedipism,” is a rare but devastating condition, often the result of acute psychosis. This condition poses a challenge to both psychiatric and ophthalmologic personnel. In the past, it was believed to be caused by either inappropriate sexual attraction to one's mother (Freud's famous Oedipus complex) or by Christian guilt after reflection on a certain passage from the Gospel of Matthew [1]. Now it is believed to result from acute schizophrenic psychosis, drug induced psychosis, obsessive-compulsive disorder, depression, or mental retardation [2, 3].

Estimated prevalence is reportedly between 2.8 and 4.2 : 100,000 and incidence is about 1 : 30 million people per year in an equal male to female ratio [2, 4]. The instrument of self-enucleation may be a finger, sharp object, or very often a usual item such as a signet ring [5]. Here, we present a case of a schizophrenic patient who attempted to enucleate his right eye using a rollerball pen.

## 2. Case Report

A 26-year-old male, diagnosed with schizophrenia six years earlier, was admitted to the Abarbanel Mental Health Center, Israel, during an acute psychotic event. While being admitted, the patient deliberately injured his right eye with a ballpoint pen. A quick response from the medical staff prevented him from self-enucleating and he was transferred to our ER for ophthalmologic evaluation. A bag containing a broken pen with a missing tip accompanied him. The patient was catatonic and noncooperative, so no history could be obtained. After administering Zyprexa 10 mg and Clonex 1 mg, we were able to examine the patient. On examination the best corrected visual acuity (BCVA) in his right eye was 20/150 and intraocular pressure (IOP) was 20 mmHg. The patient had right periorbital edema and ecchymosis, severe conjunctival chemosis, corneal erosions, and a clean anterior chamber. The pupil was dilated with no direct or consensual response to light. The vitreous was clear and fundoscopy showed a completely normal disc and retina. In addition, the patient had positive right relative afferent papillary defect (RAPD) and a complete ophthalmoplegia of the right eye. Examination of the left eye was normal, with visual acuity of 20/20 and IOP of 14 mmHg.

**Figure 1:**
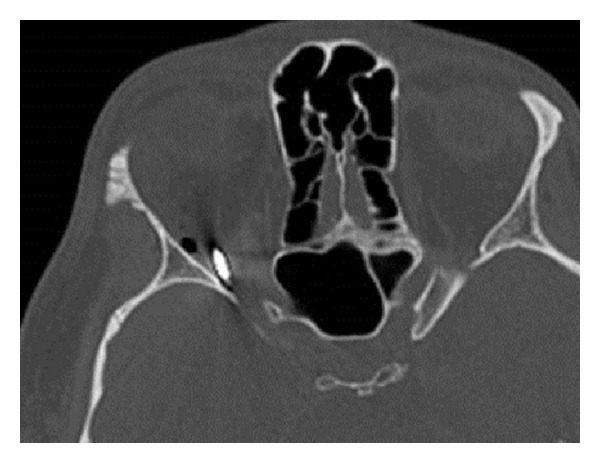
Horizontal head CT showing a metallic foreign body in the right apex.

On orbital CT there was no sign of perforation. It did show, however, a metallic foreign body in the right orbital apex close to the optic nerve and adjacent to the superior orbital fissure (Figure 1).

**Figure 2:**
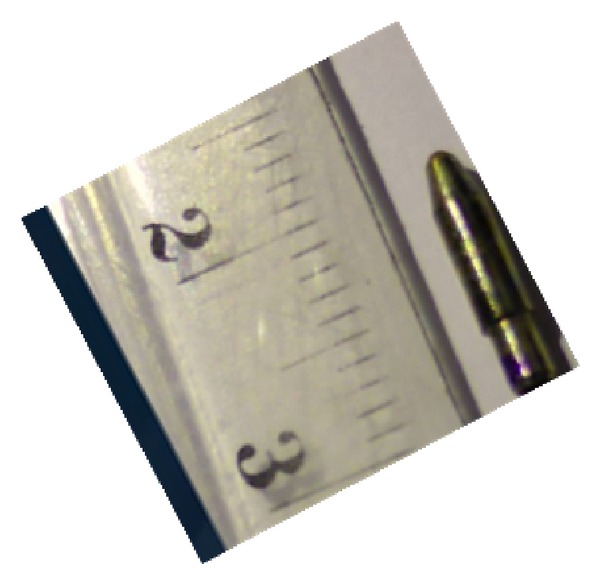
The pen tip removed from the patient's orbit.

Because the patient displayed signs of traumatic orbital apex syndrome, we planned an urgent surgery to remove the foreign body. However, it was clear that the patient, suffering from acute psychosis, is unable to give an informed consent. Therefore, the decision to operate was made and the signatures of three physicians were obtained, as dictated by the Israeli Patient's Rights Law [6]. Intravenous cefazolin and parenteral cotrimoxazole were administered to protect against aerobic and anaerobic bacteria. A lateral orbitotomy through the superior lid crease incision was performed. A 1.2-centimeter long metallic pen tip was located and retrieved from the apex (Figure 2). After removing the object, drain was left and the orbit was repaired.

**Figure 3:**
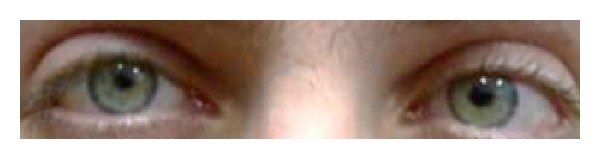
This photo was taken several seconds after testing for near accommodation. The right pupil had a normal constriction phase but later demonstrated a slower dilation response then the left pupil. This anisocoria is a result of a right tonic pupil due to ciliary ganglion injury.

The following week, there was no sign of infection and the patient was discharged with BCVA of 20/60, a dilated pupil slightly reactive to light, and complete ophthalmoplegia. Sixty-six days after surgery the visual acuity had improved to 20/20. The patient had slight ptosis, slight restriction in RE adduction with diplopia, and a pupil responsive to accommodation but only slightly responsive to light, with no RAPD (Figure 3). The patient's eye exam during his follow-up period is described in Table 1.

## 3. Discussion

Approximately eighty case reports on this rare condition exist in medical literature. Patton's 2004 review suggests several causative psychological factors [1]. In about half of the cases, religious ideation was at the root of the psychosis; the patient, preoccupied with sinful thoughts, would attack his own eye, as it symbolises the soul. Sexual ideation was found to be a cause in one-third of the cases. Some theories suggest that the act mimics autocastration and that the eye represents the penis. Others suggest that self-enucleation results from an Oedipus complex or repressed homosexuality or that it might be an autoerotic act similar to masturbation.

Regardless of the psychological origin of the self-enucleation, all of the patients who attempt it suffer from acute psychosis, often resulting from untreated schizophrenia, and have bizarre delusions about their eyes [2]. Until such severe psychosis can be brought under control using antipsychotic drugs, the patient should be restrained to keep him from hurting himself.

At presentation the patient exhibited traumatic optic neuropathy and complete ophthalmoplegia with pupillary involvement. In this case of penetrating trauma, direct damage to the cranial nerves and ocular muscles caused this orbital apex syndrome. Other complications described in reports on self-enucleation include damage to the ophthalmic artery, aneurysm of internal carotid and ophthalmic arteries, endophthalmitis, meningitis, and subarachnoid hemorrhage [1]. Our patient had a light near-dissociation response, most probably due to trauma to the ciliary ganglion, a parasympathetic ganglion located between the optic nerve and lateral rectus. Because 93% of the postciliary ganglion axons innervate the ciliary body for accommodation and only 7% innervate the iris for miosis during light reflex, pupil response to accommodation is usually spared after such injuries.

This case emphasizes the importance of collaboration between psychiatric and ophthalmic teams in order to preserve a patient's vision after a self-enucleation attempt.

## Figures and Tables

**Table 1:** Right eye exam during follow-up.

	Pre-OP	POD7	POD22	POD66
BCVA	20/150	20/60	20/50	20/20
Pupil	Dilated No direct or consensual response	Dilated Responsive Light +/−	Dilated Responsive Light +/− Accommodation ++	Dilated Responsive Light +/− Accommodation ++
RAPD	+++	Not available	+/++	Normal
EOM	No	No	No	90%

BCVA: best corrected visual acuity; EOM: extraocular movement; POD: postoperative day; OP: operative.
